# Supplementary material for: Biallelic Loss-of-Function Variant in MINPP1 Causes Pontocerebellar Hypoplasia with Characteristic Severe Neurodevelopmental Disorder
Source: Int J Mol Sci. 2025 May 29;26(11):5213. doi: 10.3390/ijms26115213 (PMC12154299; doi:10.3390/ijms26115213)
Supplement: Supplementary file 1 [file ijms-26-05213-s001.zip › ijms-3583339-Figure S1.pdf]

Supplementary Figure S1: Family pedigree and genetic confirmation of *MINPP1* variant

A. **B.**

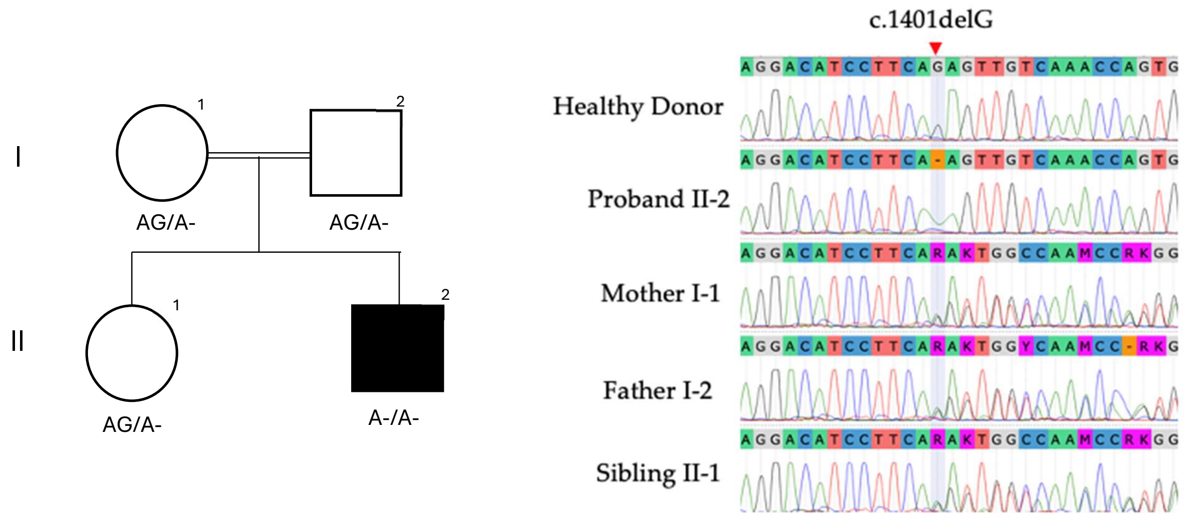

A. Family pedigree of the patient along with genotypes of the *MINPP1* variant.

B. Chromatogram of Sanger sequencing showing the variant position. The patient is homozygous for the variant; both parents and the unaffected sibling are heterozygous carriers, while the unrelated healthy donor is homozygous for the reference allele
