# Supplementary material for: Biallelic Loss-of-Function Variant in MINPP1 Causes Pontocerebellar Hypoplasia with Characteristic Severe Neurodevelopmental Disorder
Source: Int J Mol Sci. 2025 May 29;26(11):5213. doi: 10.3390/ijms26115213 (PMC12154299; doi:10.3390/ijms26115213)
Supplement: Supplementary file 1 [file ijms-26-05213-s001.zip › ijms-3583339-Table S1.pdf]

BIOCHEMICAL	Zscore	SUPER_PATHWAY	SUB_PATHWAY
glycine	0.806	Amino Acid	Glycine, Serine and Threonine Metabolism
N-acetylglycine	0.615	Amino Acid	Glycine, Serine and Threonine Metabolism
sarcosine	-2.112	Amino Acid	Glycine, Serine and Threonine Metabolism
dimethylglycine	-1.461	Amino Acid	Glycine, Serine and Threonine Metabolism
betaine	-1.208	Amino Acid	Glycine, Serine and Threonine Metabolism
serine	0.169	Amino Acid	Glycine, Serine and Threonine Metabolism
N-acetylserine	2.854	Amino Acid	Glycine, Serine and Threonine Metabolism
threonine	2.524	Amino Acid	Glycine, Serine and Threonine Metabolism
N-acetylthreonine	-0.665	Amino Acid	Glycine, Serine and Threonine Metabolism
alanine	0.874	Amino Acid	Alanine and Aspartate Metabolism
N-acetylalanine	0.493	Amino Acid	Alanine and Aspartate Metabolism
N-methylalanine	-0.603	Amino Acid	Alanine and Aspartate Metabolism
aspartate	-1.776	Amino Acid	Alanine and Aspartate Metabolism
N-acetylaspartate (NAA)	-1.971	Amino Acid	Alanine and Aspartate Metabolism
asparagine	0.208	Amino Acid	Alanine and Aspartate Metabolism
hydroxyasparagine**	0.166	Amino Acid	Alanine and Aspartate Metabolism
glutamate	-0.195	Amino Acid	Glutamate Metabolism
glutamine	1.450	Amino Acid	Glutamate Metabolism
alpha-ketoglutaramate*	0.467	Amino Acid	Glutamate Metabolism
N-acetylglutamate	-0.084	Amino Acid	Glutamate Metabolism

N-acetylglutamine	Low filled	Amino Acid	Glutamate Metabolism
4-hydroxyglutamate	-0.906	Amino Acid	Glutamate Metabolism
N-acetyl-aspartyl-glutamate (NAAG)	Not Scored	Amino Acid	Glutamate Metabolism
beta-citrylglutamate	-0.805	Amino Acid	Glutamate Metabolism
carboxyethyl-GABA	0.313	Amino Acid	Glutamate Metabolism
2-pyrrolidinone	-1.077	Amino Acid	Glutamate Metabolism
S-1-pyrroline-5-carboxylate	-0.184	Amino Acid	Glutamate Metabolism
histidine	0.291	Amino Acid	Histidine Metabolism
3-methylhistidine	-1.359	Amino Acid	Histidine Metabolism
N-acetylhistidine	-0.845	Amino Acid	Histidine Metabolism
hydantoin-5-propionate	-0.286	Amino Acid	Histidine Metabolism
trans-urocanate	-1.116	Amino Acid	Histidine Metabolism
imidazole propionate	0.422	Amino Acid	Histidine Metabolism
imidazole lactate	-1.274	Amino Acid	Histidine Metabolism
N-acetylcarnosine	0.275	Amino Acid	Histidine Metabolism
1-methyl-4-imidazoleacetate	0.513	Amino Acid	Histidine Metabolism
1-ribosyl-imidazoleacetate*	0.122	Amino Acid	Histidine Metabolism
4-imidazoleacetate	0.636	Amino Acid	Histidine Metabolism
lysine	0.955	Amino Acid	Lysine Metabolism
N6-acetyllysine	0.879	Amino Acid	Lysine Metabolism
N6,N6,N6-trimethyllysine	-0.330	Amino Acid	Lysine Metabolism
5-hydroxylysine	0.036	Amino Acid	Lysine Metabolism
5-(galactosylhydroxy)-lysine	-0.855	Amino Acid	Lysine Metabolism
2-aminoadipate	0.053	Amino Acid	Lysine Metabolism
pipecolate	-0.147	Amino Acid	Lysine Metabolism
6-oxopiperidine-2-carboxylate	1.034	Amino Acid	Lysine Metabolism
N-acetyl-cadaverine	Low filled	Amino Acid	Lysine Metabolism
N,N,N-trimethyl-5-aminovalerate	-4.549	Amino Acid	Lysine Metabolism
phenylalanine	-0.959	Amino Acid	Phenylalanine Metabolism
N-acetylphenylalanine	-0.903	Amino Acid	Phenylalanine Metabolism
phenylpyruvate	-0.230	Amino Acid	Phenylalanine Metabolism
phenyllactate (PLA)	-2.661	Amino Acid	Phenylalanine Metabolism

2-hydroxyphenylacetate	0.751	Amino Acid	Phenylalanine Metabolism
4-hydroxyphenylacetate	Not Scored	Amino Acid	Phenylalanine Metabolism
tyrosine	0.627	Amino Acid	Tyrosine Metabolism
N-acetyltyrosine	Low filled	Amino Acid	Tyrosine Metabolism
4-hydroxyphenylpyruvate	2.022	Amino Acid	Tyrosine Metabolism
3-(4-hydroxyphenyl)lactate	-0.248	Amino Acid	Tyrosine Metabolism
phenol sulfate	-0.714	Amino Acid	Tyrosine Metabolism
4-methoxyphenol sulfate	0.582	Amino Acid	Tyrosine Metabolism
vanillactate	0.766	Amino Acid	Tyrosine Metabolism
vanillylmandelate (VMA)	-0.701	Amino Acid	Tyrosine Metabolism
3-methoxytyrosine	0.379	Amino Acid	Tyrosine Metabolism
3-methoxytyramine sulfate	Low filled	Amino Acid	Tyrosine Metabolism
dopamine 4-sulfate	Not Scored	Amino Acid	Tyrosine Metabolism
dopamine 3-O-sulfate	0.468	Amino Acid	Tyrosine Metabolism
p-cresol glucuronide*	1.173	Amino Acid	Tyrosine Metabolism
tyramine O-sulfate	-0.257	Amino Acid	Tyrosine Metabolism
vanillic alcohol sulfate	1.784	Amino Acid	Tyrosine Metabolism
thyroxine	-1.024	Amino Acid	Tyrosine Metabolism
tryptophan	0.786	Amino Acid	Tryptophan Metabolism
N-acetyltryptophan	0.935	Amino Acid	Tryptophan Metabolism
C-glycosyltryptophan	-0.129	Amino Acid	Tryptophan Metabolism
kynurenine	0.118	Amino Acid	Tryptophan Metabolism
kynurenate	-0.049	Amino Acid	Tryptophan Metabolism
N-formylanthranilic acid	1.196	Amino Acid	Tryptophan Metabolism
xanthurenate	0.277	Amino Acid	Tryptophan Metabolism
picolinate	-0.507	Amino Acid	Tryptophan Metabolism
indolelactate	-0.144	Amino Acid	Tryptophan Metabolism
indoleacetate	1.086	Amino Acid	Tryptophan Metabolism

indolepropionate	-0.733	Amino Acid	Tryptophan Metabolism
indoleacetylglutamine	Not Scored	Amino Acid	Tryptophan Metabolism
indoleacetoylcarnitine*	Not Scored	Amino Acid	Tryptophan Metabolism
3-indoxyl sulfate	0.575	Amino Acid	Tryptophan Metabolism
6-bromotryptophan	-1.381	Amino Acid	Tryptophan Metabolism
leucine	0.472	Amino Acid	Leucine, Isoleucine and Valine Metabolism
N-acetylleucine	-1.243	Amino Acid	Leucine, Isoleucine and Valine Metabolism
4-methyl-2-oxopentanoate	0.565	Amino Acid	Leucine, Isoleucine and Valine Metabolism
alpha-hydroxyisocaproate	-0.248	Amino Acid	Leucine, Isoleucine and Valine Metabolism
isovalerate (i5:0)	-0.239	Amino Acid	Leucine, Isoleucine and Valine Metabolism
isovalerylglycine	0.889	Amino Acid	Leucine, Isoleucine and Valine Metabolism
isovalerylcarnitine (C5)	0.398	Amino Acid	Leucine, Isoleucine and Valine Metabolism
3-methylcrotonylglycine	Rare	Amino Acid	Leucine, Isoleucine and Valine Metabolism
beta-hydroxyisovalerate	-0.057	Amino Acid	Leucine, Isoleucine and Valine Metabolism
3-methylglutaconate	0.464	Amino Acid	Leucine, Isoleucine and Valine Metabolism
3-methylglutarylcarnitine (2)	-0.157	Amino Acid	Leucine, Isoleucine and Valine Metabolism
isoleucine	0.152	Amino Acid	Leucine, Isoleucine and Valine Metabolism
3-methyl-2-oxovalerate	0.523	Amino Acid	Leucine, Isoleucine and Valine Metabolism
2-hydroxy-3-methylvalerate	0.242	Amino Acid	Leucine, Isoleucine and Valine Metabolism
2-methylbutyrylcarnitine (C5)	0.737	Amino Acid	Leucine, Isoleucine and Valine Metabolism

tiglylcarnitine (C5:1-DC)	-1.289	Amino Acid	Leucine, Isoleucine and Valine Metabolism
3-hydroxy-2-ethylpropionate	-0.627	Amino Acid	Leucine, Isoleucine and Valine Metabolism
ethylmalonate	1.880	Amino Acid	Leucine, Isoleucine and Valine Metabolism
methylsuccinate	1.758	Amino Acid	Leucine, Isoleucine and Valine Metabolism
valine	-0.325	Amino Acid	Leucine, Isoleucine and Valine Metabolism
N-acetylvaline	-0.513	Amino Acid	Leucine, Isoleucine and Valine Metabolism
3-methyl-2-oxobutyrate	-0.407	Amino Acid	Leucine, Isoleucine and Valine Metabolism
alpha-hydroxyisovalerate	-1.347	Amino Acid	Leucine, Isoleucine and Valine Metabolism
isobutyrylcarnitine (C4)	0.517	Amino Acid	Leucine, Isoleucine and Valine Metabolism
isobutyrylglycine	1.292	Amino Acid	Leucine, Isoleucine and Valine Metabolism
3-hydroxyisobutyrate	-0.521	Amino Acid	Leucine, Isoleucine and Valine Metabolism
methionine	-0.089	Amino Acid	Methionine, Cysteine, SAM and Taurine Metabolism
N-acetylmethionine	0.467	Amino Acid	Methionine, Cysteine, SAM and Taurine Metabolism
N-formylmethionine	0.156	Amino Acid	Methionine, Cysteine, SAM and Taurine Metabolism
methionine sulfone	2.068	Amino Acid	Methionine, Cysteine, SAM and Taurine Metabolism
methionine sulfoxide	1.945	Amino Acid	Methionine, Cysteine, SAM and Taurine Metabolism
cystathionine	Not Scored	Amino Acid	Methionine, Cysteine, SAM and Taurine Metabolism

alpha-ketobutyrate	-0.349	Amino Acid	Methionine, Cysteine, SAM and Taurine Metabolism
cysteine	2.360	Amino Acid	Methionine, Cysteine, SAM and Taurine Metabolism
S-methylcysteine	-0.255	Amino Acid	Methionine, Cysteine, SAM and Taurine Metabolism
S-methylcysteine sulfoxide	-0.331	Amino Acid	Methionine, Cysteine, SAM and Taurine Metabolism
cysteine s-sulfate	-0.386	Amino Acid	Methionine, Cysteine, SAM and Taurine Metabolism
cystine	-0.014	Amino Acid	Methionine, Cysteine, SAM and Taurine Metabolism
hypotaurine	-1.842	Amino Acid	Methionine, Cysteine, SAM and Taurine Metabolism
taurine	-0.512	Amino Acid	Methionine, Cysteine, SAM and Taurine Metabolism
N-acetyltaurine	0.910	Amino Acid	Methionine, Cysteine, SAM and Taurine Metabolism
arginine	0.122	Amino Acid	Urea cycle; Arginine and Proline Metabolism
argininosuccinate	Rare	Amino Acid	Urea cycle; Arginine and Proline Metabolism
urea	-0.545	Amino Acid	Urea cycle; Arginine and Proline Metabolism
ornithine	-0.824	Amino Acid	Urea cycle; Arginine and Proline Metabolism
2-oxoarginine*	0.476	Amino Acid	Urea cycle; Arginine and Proline Metabolism

citrulline	0.024	Amino Acid	Urea cycle; Arginine and Proline Metabolism
homoarginine	-0.571	Amino Acid	Urea cycle; Arginine and Proline Metabolism
homocitrulline	1.952	Amino Acid	Urea cycle; Arginine and Proline Metabolism
proline	0.416	Amino Acid	Urea cycle; Arginine and Proline Metabolism
dimethylarginine (SDMA + ADMA)	0.637	Amino Acid	Urea cycle; Arginine and Proline Metabolism
N-acetylarginine	-1.983	Amino Acid	Urea cycle; Arginine and Proline Metabolism
N-acetylcitrulline	0.132	Amino Acid	Urea cycle; Arginine and Proline Metabolism
N-delta-acetylornithine	0.751	Amino Acid	Urea cycle; Arginine and Proline Metabolism
N-alpha-acetylornithine	2.098	Amino Acid	Urea cycle; Arginine and Proline Metabolism
trans-4-hydroxyproline	0.117	Amino Acid	Urea cycle; Arginine and Proline Metabolism
pro-hydroxy-pro	0.131	Amino Acid	Urea cycle; Arginine and Proline Metabolism
N-methylproline	-2.275	Amino Acid	Urea cycle; Arginine and Proline Metabolism
argininate*	-0.624	Amino Acid	Urea cycle; Arginine and Proline Metabolism
guanidinoacetate	-1.062	Amino Acid	Creatine Metabolism
creatine	-0.910	Amino Acid	Creatine Metabolism

creatinine	-1.208	Amino Acid	Creatine Metabolism
N-acetylputrescine	0.828	Amino Acid	Polyamine Metabolism
N-acetyl-isoputreanine	-0.350	Amino Acid	Polyamine Metabolism
spermidine	-1.026	Amino Acid	Polyamine Metabolism
(N(1) + N(8))-acetylspermidine	0.482	Amino Acid	Polyamine Metabolism
5-methylthioadenosine (MTA)	Low filled	Amino Acid	Polyamine Metabolism
4-acetamidobutanoate	-0.232	Amino Acid	Polyamine Metabolism
4-guanidinobutanoate	-2.172	Amino Acid	Guanidino and Acetamido Metabolism
cysteinylglycine	1.732	Amino Acid	Glutathione Metabolism
cysteinylglycine disulfide*	0.700	Amino Acid	Glutathione Metabolism
cys-gly, oxidized	0.448	Amino Acid	Glutathione Metabolism
5-oxoproline	-0.830	Amino Acid	Glutathione Metabolism
2-aminobutyrate	-1.012	Amino Acid	Glutathione Metabolism
2-hydroxybutyrate/2-hydroxyisobutyrate	-0.478	Amino Acid	Glutathione Metabolism
gamma-glutamylglutamate	-0.378	Peptide	Gamma-glutamyl Amino Acid
gamma-glutamylglutamine	0.840	Peptide	Gamma-glutamyl Amino Acid
gamma-glutamylglycine	-0.261	Peptide	Gamma-glutamyl Amino Acid
gamma-glutamylhistidine	-0.398	Peptide	Gamma-glutamyl Amino Acid
gamma-glutamylisoleucine*	-0.465	Peptide	Gamma-glutamyl Amino Acid
gamma-glutamylleucine	-0.176	Peptide	Gamma-glutamyl Amino Acid
gamma-glutamyl-alpha-lysine	-0.059	Peptide	Gamma-glutamyl Amino Acid
gamma-glutamyl-epsilon-lysine	0.501	Peptide	Gamma-glutamyl Amino Acid
gamma-glutamylmethionine	-0.348	Peptide	Gamma-glutamyl Amino Acid
gamma-glutamylphenylalanine	-1.022	Peptide	Gamma-glutamyl Amino Acid
gamma-glutamylthreonine	1.780	Peptide	Gamma-glutamyl Amino Acid
gamma-glutamyltryptophan	0.351	Peptide	Gamma-glutamyl Amino Acid
gamma-glutamyltyrosine	0.054	Peptide	Gamma-glutamyl Amino Acid

gamma-glutamylvaline	-0.523	Peptide	Gamma-glutamyl Amino Acid
gamma-glutamylcitrulline*	Not Scored	Peptide	Gamma-glutamyl Amino Acid
isoleucylglycine	-0.655	Peptide	Dipeptide
leucylglycine	-0.370	Peptide	Dipeptide
leucylleucine	-0.040	Peptide	Dipeptide
isoleucylleucine/leucylisoleucine	-0.040	Peptide	Dipeptide
fibrinopeptide A*	0.938	Peptide	Fibrinogen Cleavage Peptide
fibrinopeptide A, des-ala(1)*	1.051	Peptide	Fibrinogen Cleavage Peptide
phenylacetylcarnitine	-0.171	Peptide	Acetylated Peptides
phenylacetylglutamine	1.118	Peptide	Acetylated Peptides
1,5-anhydroglucitol (1,5-AG)	-0.222	Carbohydrate	Glycolysis, Gluconeogenesis, and Pyruvate Metabolism
glucose	-0.068	Carbohydrate	Glycolysis, Gluconeogenesis, and Pyruvate Metabolism
3-phosphoglycerate	-1.897	Carbohydrate	Glycolysis, Gluconeogenesis, and Pyruvate Metabolism
pyruvate	0.344	Carbohydrate	Glycolysis, Gluconeogenesis, and Pyruvate Metabolism
lactate	0.374	Carbohydrate	Glycolysis, Gluconeogenesis, and Pyruvate Metabolism
glycerate	-0.314	Carbohydrate	Glycolysis, Gluconeogenesis, and Pyruvate Metabolism
ribitol	1.345	Carbohydrate	Pentose Metabolism
ribonate	-0.670	Carbohydrate	Pentose Metabolism
xylose	-0.815	Carbohydrate	Pentose Metabolism
arabinose	Low filled	Carbohydrate	Pentose Metabolism
arabitol/xylitol	2.640	Carbohydrate	Pentose Metabolism
arabonate/xylonate	-1.224	Carbohydrate	Pentose Metabolism
lyxonate	-0.281	Carbohydrate	Pentose Metabolism
maltose	-0.038	Carbohydrate	Glycogen Metabolism

sucrose	1.024	Carbohydrate	Disaccharides and Oligosaccharides
fructose	-0.832	Carbohydrate	Fructose, Mannose and Galactose Metabolism
mannitol/sorbitol	2.161	Carbohydrate	Fructose, Mannose and Galactose Metabolism
mannose	Not Scored	Carbohydrate	Fructose, Mannose and Galactose Metabolism
glucuronate	1.810	Carbohydrate	Aminosugar Metabolism
N-acetylneuraminate	-0.264	Carbohydrate	Aminosugar Metabolism
erythronate*	-0.312	Carbohydrate	Aminosugar Metabolism
N-acetylglucosamine/N-acetylgalactosamine	-1.004	Carbohydrate	Aminosugar Metabolism
N6-carboxymethyllysine	Low filled	Carbohydrate	Advanced Glycation End-product
citrate	-1.365	Energy	TCA Cycle
aconitate [cis or trans]	-0.827	Energy	TCA Cycle
isocitric lactone	0.844	Energy	TCA Cycle
alpha-ketoglutarate	0.978	Energy	TCA Cycle
succinylcarnitine (C4-DC)	-0.230	Energy	TCA Cycle
succinate	-0.690	Energy	TCA Cycle
fumarate	1.447	Energy	TCA Cycle
malate	0.461	Energy	TCA Cycle
phosphate	1.270	Energy	Oxidative Phosphorylation
malonylcarnitine	Low filled	Lipid	Fatty Acid Synthesis
malonate	-1.576	Lipid	Fatty Acid Synthesis
caproate (6:0)	-1.484	Lipid	Medium/Long Chain Fatty Acid
heptanoate (7:0)	1.894	Lipid	Medium/Long Chain Fatty Acid
caprylate (8:0)	2.213	Lipid	Medium/Long Chain Fatty Acid
caprate (10:0)	1.517	Lipid	Medium/Long Chain Fatty Acid
10-undecenoate (11:1n1)	-2.064	Lipid	Medium/Long Chain Fatty Acid
laurate (12:0)	-1.847	Lipid	Medium/Long Chain Fatty Acid
5-dodecenoate (12:1n7)	-3.070	Lipid	Medium/Long Chain Fatty Acid
myristate (14:0)	-1.636	Lipid	Medium/Long Chain Fatty Acid
palmitate (16:0)	-0.837	Lipid	Medium/Long Chain Fatty Acid

margarate (17:0)	-1.141	Lipid	Medium/Long Chain Fatty Acid
stearate (18:0)	-0.619	Lipid	Medium/Long Chain Fatty Acid
nonadecanoate (19:0)	-0.662	Lipid	Medium/Long Chain Fatty Acid
arachidate (20:0)	-0.307	Lipid	Medium/Long Chain Fatty Acid
behenate (22:0)*	0.328	Lipid	Medium/Long Chain Fatty Acid
myristoleate (14:1n5)	-2.814	Lipid	Medium/Long Chain Fatty Acid
palmitoleate (16:1n7)	-2.053	Lipid	Medium/Long Chain Fatty Acid
10-heptadecenoate (17:1n7)	-1.971	Lipid	Medium/Long Chain Fatty Acid
oleate/vaccenate (18:1)	-2.453	Lipid	Medium/Long Chain Fatty Acid
10-nonadecenoate (19:1n9)	-2.891	Lipid	Medium/Long Chain Fatty Acid
eicosenoate (20:1)	-1.853	Lipid	Medium/Long Chain Fatty Acid
erucate (22:1n9)	-0.447	Lipid	Medium/Long Chain Fatty Acid
stearidonate (18:4n3)	-0.818	Lipid	Medium/Long Chain Fatty Acid
eicosapentaenoate (EPA; 20:5n3)	-0.052	Lipid	Medium/Long Chain Fatty Acid
docosapentaenoate (n3 DPA; 22:5n3)	-1.059	Lipid	Medium/Long Chain Fatty Acid
docosahexaenoate (DHA; 22:6n3)	-1.744	Lipid	Medium/Long Chain Fatty Acid
docosatrienoate (22:3n3)	-0.190	Lipid	Medium/Long Chain Fatty Acid
hexadecadienoate (16:2n6)	-2.938	Lipid	Medium/Long Chain Fatty Acid
linoleate (18:2n6)	-2.636	Lipid	Medium/Long Chain Fatty Acid
linolenate [alpha or gamma; (18:3n3 or 6)]	-1.704	Lipid	Medium/Long Chain Fatty Acid
dihomo-linoleate (20:2n6)	-1.672	Lipid	Medium/Long Chain Fatty Acid
dihomo-linolenate (20:3n3 or n6)	-0.361	Lipid	Medium/Long Chain Fatty Acid
arachidonate (20:4n6)	-1.715	Lipid	Medium/Long Chain Fatty Acid
adrenate (22:4n6)	-2.173	Lipid	Medium/Long Chain Fatty Acid
docosapentaenoate (n6 DPA; 22:5n6)	-1.405	Lipid	Medium/Long Chain Fatty Acid
docosadienoate (22:2n6)	-0.981	Lipid	Medium/Long Chain Fatty Acid
(14 or 15)-methylpalmitate (a17:0 or i17:0)	-1.509	Lipid	Fatty Acid, Branched
(16 or 17)-methylstearate (a19:0 or i19:0)	-1.150	Lipid	Fatty Acid, Branched

glutarate (C5-DC)	-2.079	Lipid	Fatty Acid, Dicarboxylate
2-hydroxyglutarate	0.231	Lipid	Fatty Acid, Dicarboxylate
4-hydroxy-2-oxoglutaric acid	0.925	Lipid	Fatty Acid, Dicarboxylate
3-hydroxyadipate	-0.179	Lipid	Fatty Acid, Dicarboxylate
maleate	1.831	Lipid	Fatty Acid, Dicarboxylate
suberate (C8-DC)	-0.222	Lipid	Fatty Acid, Dicarboxylate
azelate (C9-DC)	-1.703	Lipid	Fatty Acid, Dicarboxylate
sebacate (C10-DC)	1.726	Lipid	Fatty Acid, Dicarboxylate
dodecanedioate (C12-DC)	-2.170	Lipid	Fatty Acid, Dicarboxylate
dodecadienoate (12:2)*	-3.871	Lipid	Fatty Acid, Dicarboxylate
tetradecanedioate (C14-DC)	-1.795	Lipid	Fatty Acid, Dicarboxylate
hexadecanedioate (C16-DC)	-1.328	Lipid	Fatty Acid, Dicarboxylate
hexadecenedioate (C16:1-DC)*	-2.751	Lipid	Fatty Acid, Dicarboxylate
octadecanedioate (C18-DC)	-3.008	Lipid	Fatty Acid, Dicarboxylate
octadecenedioate (C18:1-DC)	-3.409	Lipid	Fatty Acid, Dicarboxylate
octadecadienedioate (C18:2-DC)*	-1.154	Lipid	Fatty Acid, Dicarboxylate
eicosanedioate (C20-DC)	-1.082	Lipid	Fatty Acid, Dicarboxylate
docosadioate (C22-DC)	0.315	Lipid	Fatty Acid, Dicarboxylate
3-carboxy-4-methyl-5-propyl-2-furanpropanoate (CMPF)	-1.601	Lipid	Fatty Acid, Dicarboxylate
hydroxy-CMPF*	-0.827	Lipid	Fatty Acid, Dicarboxylate
3-carboxy-4-methyl-5-pentyl-2-furanpropionate (3-CMPFP)**	-4.776	Lipid	Fatty Acid, Dicarboxylate
2-aminoheptanoate	-0.578	Lipid	Fatty Acid, Amino
2-aminooctanoate	0.305	Lipid	Fatty Acid, Amino
N-acetyl-2-aminooctanoate*	-2.003	Lipid	Fatty Acid, Amino
butyrylcarnitine (C4)	1.592	Lipid	Fatty Acid Metabolism (also BCAA Metabolism)
propionylcarnitine (C3)	-0.106	Lipid	Fatty Acid Metabolism (also BCAA Metabolism)
propionylglycine	1.481	Lipid	Fatty Acid Metabolism (also BCAA Metabolism)

methylmalonate (MMA)	-1.413	Lipid	Fatty Acid Metabolism (also BCAA Metabolism)
hexanoylglutamine	2.512	Lipid	Fatty Acid Metabolism (Acyl Glutamine)
hexanoylglycine	Low filled	Lipid	Fatty Acid Metabolism (Acyl Glycine)
trans-2-hexenoylglycine	Not Scored	Lipid	Fatty Acid Metabolism (Acyl Glycine)
N-octanoylglycine	Rare	Lipid	Fatty Acid Metabolism (Acyl Glycine)
N-palmitoylglycine	-0.527	Lipid	Fatty Acid Metabolism (Acyl Glycine)
3-hydroxybutyroylglycine**	-0.122	Lipid	Fatty Acid Metabolism (Acyl Glycine)
acetylcarnitine (C2)	0.149	Lipid	Fatty Acid Metabolism (Acyl Carnitine)
hexanoylcarnitine (C6)	1.216	Lipid	Fatty Acid Metabolism (Acyl Carnitine)
octanoylcarnitine (C8)	-0.857	Lipid	Fatty Acid Metabolism (Acyl Carnitine)
decanoylcarnitine (C10)	-1.290	Lipid	Fatty Acid Metabolism (Acyl Carnitine)
laurylcarnitine (C12)	-2.624	Lipid	Fatty Acid Metabolism (Acyl Carnitine)
myristoylcarnitine (C14)	-1.220	Lipid	Fatty Acid Metabolism (Acyl Carnitine)
palmitoylcarnitine (C16)	-0.190	Lipid	Fatty Acid Metabolism (Acyl Carnitine)
margaroylcarnitine (C17)*	-0.441	Lipid	Fatty Acid Metabolism (Acyl Carnitine)
stearoylcarnitine (C18)	-0.722	Lipid	Fatty Acid Metabolism (Acyl Carnitine)
behenoylcarnitine (C22)*	-0.953	Lipid	Fatty Acid Metabolism (Acyl Carnitine)
lignoceroylcarnitine (C24)*	-1.876	Lipid	Fatty Acid Metabolism (Acyl Carnitine)
cerotoylcarnitine (C26)*	-0.026	Lipid	Fatty Acid Metabolism (Acyl Carnitine)

cis-4-decenoylcarnitine (C10:1)	-3.017	Lipid	Fatty Acid Metabolism (Acyl Carnitine)
5-dodecenoylcarnitine (C12:1)	-1.980	Lipid	Fatty Acid Metabolism (Acyl Carnitine)
myristoleoylcarnitine (C14:1)*	-2.381	Lipid	Fatty Acid Metabolism (Acyl Carnitine)
palmitoleoylcarnitine (C16:1)*	-1.098	Lipid	Fatty Acid Metabolism (Acyl Carnitine)
oleoylcarnitine (C18:1)	0.523	Lipid	Fatty Acid Metabolism (Acyl Carnitine)
eicosenoylcarnitine (C20:1)*	0.745	Lipid	Fatty Acid Metabolism (Acyl Carnitine)
nervonoylcarnitine (C24:1)*	-0.273	Lipid	Fatty Acid Metabolism (Acyl Carnitine)
ximenoylcarnitine (C26:1)*	0.780	Lipid	Fatty Acid Metabolism (Acyl Carnitine)
linoleoylcarnitine (C18:2)*	0.452	Lipid	Fatty Acid Metabolism (Acyl Carnitine)
linolenoylcarnitine (C18:3)*	1.071	Lipid	Fatty Acid Metabolism (Acyl Carnitine)
dihomo-linoleoylcarnitine (C20:2)*	1.482	Lipid	Fatty Acid Metabolism (Acyl Carnitine)
arachidonoylcarnitine (C20:4)	1.436	Lipid	Fatty Acid Metabolism (Acyl Carnitine)
dihomo-linolenoylcarnitine (C20:3n3 or 6)*	2.828	Lipid	Fatty Acid Metabolism (Acyl Carnitine)
adipoylcarnitine (C6-DC)	0.228	Lipid	Fatty Acid Metabolism (Acyl Carnitine)
pimeloylcarnitine/3-methyladipoylcarnitine (C7-DC)	Not Scored	Lipid	Fatty Acid Metabolism (Acyl Carnitine)
suberoylcarnitine (C8-DC)	0.101	Lipid	Fatty Acid Metabolism (Acyl Carnitine)
octadecanedioylcarnitine (C18-DC)*	-3.180	Lipid	Fatty Acid Metabolism (Acyl Carnitine)
octadecenedioylcarnitine (C18:1-DC)*	-2.642	Lipid	Fatty Acid Metabolism (Acyl Carnitine)
(R)-3-hydroxybutyrylcarnitine	Low filled	Lipid	Fatty Acid Metabolism (Acyl Carnitine)

(S)-3-hydroxybutyrylcarnitine	-0.309	Lipid	Fatty Acid Metabolism (Acyl Carnitine)
deoxycarnitine	-1.308	Lipid	Carnitine Metabolism
carnitine	0.612	Lipid	Carnitine Metabolism
3-hydroxybutyrate (BHBA)	-0.633	Lipid	Ketone Bodies
palmitoylcholine	1.245	Lipid	Fatty Acid Metabolism (Acyl Choline)
oleoylcholine	Not Scored	Lipid	Fatty Acid Metabolism (Acyl Choline)
linoleoylcholine*	0.991	Lipid	Fatty Acid Metabolism (Acyl Choline)
stearoylcholine*	Not Scored	Lipid	Fatty Acid Metabolism (Acyl Choline)
arachidonoylcholine	1.410	Lipid	Fatty Acid Metabolism (Acyl Choline)
alpha-hydroxycaproate	Not Scored	Lipid	Fatty Acid, Monohydroxy
2-hydroxyoctanoate	-0.459	Lipid	Fatty Acid, Monohydroxy
2-hydroxydecanoate	-0.592	Lipid	Fatty Acid, Monohydroxy
2-hydroxypalmitate	0.020	Lipid	Fatty Acid, Monohydroxy
2-hydroxystearate	0.310	Lipid	Fatty Acid, Monohydroxy
2-hydroxynervonate*	1.236	Lipid	Fatty Acid, Monohydroxy
3-hydroxyhexanoate	-1.160	Lipid	Fatty Acid, Monohydroxy
3-hydroxyoctanoate	-2.525	Lipid	Fatty Acid, Monohydroxy
3-hydroxydecanoate	-2.719	Lipid	Fatty Acid, Monohydroxy
3-hydroxylaurate	-2.967	Lipid	Fatty Acid, Monohydroxy
5-hydroxyhexanoate	Not Scored	Lipid	Fatty Acid, Monohydroxy
16-hydroxypalmitate	0.312	Lipid	Fatty Acid, Monohydroxy
13-HODE + 9-HODE	-3.703	Lipid	Fatty Acid, Monohydroxy
12,13-DiHOME	-0.336	Lipid	Fatty Acid, Dihydroxy
9,10-DiHOME	-0.942	Lipid	Fatty Acid, Dihydroxy
oleoyl ethanolamide	0.293	Lipid	Endocannabinoid
palmitoyl ethanolamide	-1.658	Lipid	Endocannabinoid
stearoyl ethanolamide	-0.916	Lipid	Endocannabinoid

N-oleoylserine	Low filled	Lipid	Endocannabinoid
N-palmitoylserine	Not Scored	Lipid	Endocannabinoid
myo-inositol	-1.067	Lipid	Inositol Metabolism
choline	-1.129	Lipid	Phospholipid Metabolism
choline phosphate	-0.650	Lipid	Phospholipid Metabolism
glycerophosphorylcholine (GPC)	-0.269	Lipid	Phospholipid Metabolism
phosphoethanolamine	-0.182	Lipid	Phospholipid Metabolism
glycerophosphoethanolamine	-0.394	Lipid	Phospholipid Metabolism
trimethylamine N-oxide	-0.489	Lipid	Phospholipid Metabolism
phosphatidylcholine (14:0/14:0, 16:0/12:0)	1.908	Lipid	Phosphatidylcholine (PC)
1-myristoyl-2-palmitoyl-GPC (14:0/16:0)	1.241	Lipid	Phosphatidylcholine (PC)
1-myristoyl-2-linoleoyl-GPC (14:0/18:2)*	-0.519	Lipid	Phosphatidylcholine (PC)
1-myristoyl-2-arachidonoyl-GPC (14:0/20:4)*	1.004	Lipid	Phosphatidylcholine (PC)
1-myristoyl-2-docosahexaenoyl-GPC (14:0/22:6)*	0.038	Lipid	Phosphatidylcholine (PC)
phosphatidylcholine (15:0/18:1, 17:0/16:1, 16:0/17:1)*	0.959	Lipid	Phosphatidylcholine (PC)
1-pentadecanoyl-2-linoleoyl-GPC (15:0/18:2)*	-1.119	Lipid	Phosphatidylcholine (PC)
1-pentadecanoyl-2-arachidonoyl-GPC (15:0/20:4)*	-0.700	Lipid	Phosphatidylcholine (PC)
1-pentadecanoyl-2-docosahexaenoyl-GPC (15:0/22:6)*	-1.516	Lipid	Phosphatidylcholine (PC)
1-palmitoyl-2-pentadecanoyl-GPC (16:0/15:0)*	-0.164	Lipid	Phosphatidylcholine (PC)
1,2-dipalmitoyl-GPC (16:0/16:0)	0.380	Lipid	Phosphatidylcholine (PC)
1-palmitoyl-2-palmitoleoyl-GPC (16:0/16:1)*	1.573	Lipid	Phosphatidylcholine (PC)
1-palmitoyl-2-stearoyl-GPC (16:0/18:0)	-0.326	Lipid	Phosphatidylcholine (PC)
1-palmitoyl-2-oleoyl-GPC (16:0/18:1)	1.734	Lipid	Phosphatidylcholine (PC)
1-palmitoyl-2-linoleoyl-GPC (16:0/18:2)	-1.138	Lipid	Phosphatidylcholine (PC)
1-palmitoyl-2-arachidonoyl-GPC (16:0/20:4n6)	-0.703	Lipid	Phosphatidylcholine (PC)
1-palmitoyl-2-eicosapentaenoyl-GPC (16:0/20:5)*	2.089	Lipid	Phosphatidylcholine (PC)
1-palmitoyl-2-adrenoyl-GPC (16:0/22:4)*	3.182	Lipid	Phosphatidylcholine (PC)
phosphatidylcholine (16:0/22:5n3, 18:1/20:4)*	0.993	Lipid	Phosphatidylcholine (PC)
1-palmitoyl-2-docosahexaenoyl-GPC (16:0/22:6)	-1.583	Lipid	Phosphatidylcholine (PC)

1-palmitoleoyl-2-linolenoyl-GPC (16:1/18:3)*	1.781	Lipid	Phosphatidylcholine (PC)
1-margaroyl-2-oleoyl-GPC (17:0/18:1)*	1.843	Lipid	Phosphatidylcholine (PC)
1-margaroyl-2-linoleoyl-GPC (17:0/18:2)*	-0.592	Lipid	Phosphatidylcholine (PC)
1-margaroyl-2-arachidonoyl-GPC (17:0/20:4)*	-1.683	Lipid	Phosphatidylcholine (PC)
1-stearoyl-2-oleoyl-GPC (18:0/18:1)	3.495	Lipid	Phosphatidylcholine (PC)
1-stearoyl-2-linoleoyl-GPC (18:0/18:2)*	-0.679	Lipid	Phosphatidylcholine (PC)
phosphatidylcholine (18:0/20:2, 20:0/18:2)*	3.791	Lipid	Phosphatidylcholine (PC)
1-stearoyl-2-dihomo-linolenoyl-GPC (18:0/20:3n3 or 6)*	2.988	Lipid	Phosphatidylcholine (PC)
1-stearoyl-2-meadoyl-GPC (18:0/20:3n9)*	Low filled	Lipid	Phosphatidylcholine (PC)
1-stearoyl-2-arachidonoyl-GPC (18:0/20:4)	-0.083	Lipid	Phosphatidylcholine (PC)
phosphatidylcholine (18:0/20:5, 16:0/22:5n6)*	2.463	Lipid	Phosphatidylcholine (PC)
1-stearoyl-2-adrenoyl-GPC (18:0/22:4)*	2.010	Lipid	Phosphatidylcholine (PC)
1-stearoyl-2-docosapentaenoyl-GPC (18:0/22:5n3)*	2.544	Lipid	Phosphatidylcholine (PC)
1-stearoyl-2-docosapentaenoyl-GPC (18:0/22:5n6)*	0.284	Lipid	Phosphatidylcholine (PC)
1-stearoyl-2-docosahexaenoyl-GPC (18:0/22:6)	-0.125	Lipid	Phosphatidylcholine (PC)
1-oleoyl-2-dihomo-linoleoyl-GPC (18:1/20:2)*	1.937	Lipid	Phosphatidylcholine (PC)
1-oleoyl-2-dihomo-linolenoyl-GPC (18:1/20:3)*	2.744	Lipid	Phosphatidylcholine (PC)
1-oleoyl-2-docosahexaenoyl-GPC (18:1/22:6)*	-0.478	Lipid	Phosphatidylcholine (PC)
1,2-dilinoleoyl-GPC (18:2/18:2)	0.169	Lipid	Phosphatidylcholine (PC)
1-linoleoyl-2-linolenoyl-GPC (18:2/18:3)*	1.813	Lipid	Phosphatidylcholine (PC)
1-linoleoyl-2-docosahexaenoyl-GPC (18:2/22:6)*	0.851	Lipid	Phosphatidylcholine (PC)
1-arachidoyl-2-arachidonoyl-GPC (20:0/20:4)*	0.213	Lipid	Phosphatidylcholine (PC)
1-lignoceroyl-2-arachidonoyl-GPC (24:0/20:4)*	0.050	Lipid	Phosphatidylcholine (PC)
1-nervonoyl-2-arachidonoyl-GPC (24:1/20:4)*	0.863	Lipid	Phosphatidylcholine (PC)
1-palmitoyl-2-oleoyl-GPE (16:0/18:1)	1.472	Lipid	Phosphatidylethanolamine (PE)
1-palmitoyl-2-linoleoyl-GPE (16:0/18:2)	0.575	Lipid	Phosphatidylethanolamine (PE)
1-palmitoyl-2-arachidonoyl-GPE (16:0/20:4)*	-0.412	Lipid	Phosphatidylethanolamine (PE)
1-palmitoyl-2-docosahexaenoyl-GPE (16:0/22:6)*	-1.466	Lipid	Phosphatidylethanolamine (PE)

1-stearoyl-2-oleoyl-GPE (18:0/18:1)	0.878	Lipid	Phosphatidylethanolamine (PE)
1-stearoyl-2-linoleoyl-GPE (18:0/18:2)*	0.849	Lipid	Phosphatidylethanolamine (PE)
1-stearoyl-2-dihomo-linolenoyl-GPE (18:0/20:3n3 or 6)*	1.605	Lipid	Phosphatidylethanolamine (PE)
1-stearoyl-2-arachidonoyl-GPE (18:0/20:4)	-0.779	Lipid	Phosphatidylethanolamine (PE)
1-stearoyl-2-adrenoyl-GPE (18:0/22:4)*	0.066	Lipid	Phosphatidylethanolamine (PE)
1-stearoyl-2-docosahexaenoyl-GPE (18:0/22:6)*	-0.380	Lipid	Phosphatidylethanolamine (PE)
1-oleoyl-2-linoleoyl-GPE (18:1/18:2)*	0.992	Lipid	Phosphatidylethanolamine (PE)
phosphatidylethanolamine (P-18:1/20:4, P-16:0/22:5n3)*	-2.178	Lipid	Phosphatidylethanolamine (PE)
1,2-dilinoleoyl-GPE (18:2/18:2)*	0.718	Lipid	Phosphatidylethanolamine (PE)
1-stearoyl-2-oleoyl-GPS (18:0/18:1)	-1.574	Lipid	Phosphatidylserine (PS)
1-stearoyl-2-linoleoyl-GPI (18:0/18:2)	0.540	Lipid	Phosphatidylinositol (PI)
1-stearoyl-2-arachidonoyl-GPI (18:0/20:4)	-1.496	Lipid	Phosphatidylinositol (PI)
1-stearoyl-2-dihomo-linolenoyl-GPI (18:0/20:3n3 or 6)*	2.075	Lipid	Phosphatidylinositol (PI)
1-palmitoyl-GPA (16:0)	-1.367	Lipid	Lysophospholipid
1-linoleoyl-GPA (18:2)*	-2.659	Lipid	Lysophospholipid
1-arachidonoyl-GPA (20:4)	-1.309	Lipid	Lysophospholipid
1-myristoyl-GPC (14:0)	0.698	Lipid	Lysophospholipid
1-pentadecanoyl-GPC (15:0)*	0.145	Lipid	Lysophospholipid
1-palmitoyl-GPC (16:0)	0.735	Lipid	Lysophospholipid
2-palmitoyl-GPC (16:0)*	0.813	Lipid	Lysophospholipid
1-palmitoleoyl-GPC (16:1)*	1.549	Lipid	Lysophospholipid
2-palmitoleoyl-GPC (16:1)*	Low filled	Lipid	Lysophospholipid
1-margaroyl-GPC (17:0)	0.378	Lipid	Lysophospholipid
1-stearoyl-GPC (18:0)	1.221	Lipid	Lysophospholipid
2-stearoyl-GPC (18:0)*	0.434	Lipid	Lysophospholipid
1-oleoyl-GPC (18:1)	2.146	Lipid	Lysophospholipid
2-oleoyl-GPC (18:1)*	1.976	Lipid	Lysophospholipid
1-linoleoyl-GPC (18:2)	0.885	Lipid	Lysophospholipid
2-linoleoyl-GPC (18:2)*	0.279	Lipid	Lysophospholipid
1-linolenoyl-GPC (18:3)*	2.670	Lipid	Lysophospholipid
1-nonadecanoyl-GPC (19:0)	1.003	Lipid	Lysophospholipid
1-arachidoyl-GPC (20:0)	1.513	Lipid	Lysophospholipid
1-eicosenoyl-GPC (20:1)*	1.644	Lipid	Lysophospholipid
1-dihomo-linoleoyl-GPC (20:2)*	2.292	Lipid	Lysophospholipid
1-dihomo-linolenoyl-GPC (20:3n3 or 6)*	2.547	Lipid	Lysophospholipid
2-arachidonoyl-GPC (20:4)*	0.028	Lipid	Lysophospholipid
1-arachidonoyl-GPC (20:4n6)*	0.210	Lipid	Lysophospholipid

1-eicosapentaenoyl-GPC (20:5)*	2.064	Lipid	Lysophospholipid
1-behenoyl-GPC (22:0)	0.336	Lipid	Lysophospholipid
1-erucoyl-GPC (22:1)*	1.859	Lipid	Lysophospholipid
1-adrenoyl-GPC (22:4)*	0.986	Lipid	Lysophospholipid
1-docosapentaenoyl-GPC (22:5n3)*	1.692	Lipid	Lysophospholipid
1-docosapentaenoyl-GPC (22:5n6)*	0.211	Lipid	Lysophospholipid
1-docosahexaenoyl-GPC (22:6)*	-0.065	Lipid	Lysophospholipid
1-lignoceroyl-GPC (24:0)	1.673	Lipid	Lysophospholipid
1-nervonoyl-GPC (24:1n9)*	Low filled	Lipid	Lysophospholipid
1-cerotoyl-GPC (26:0)*	Low filled	Lipid	Lysophospholipid
1-palmitoyl-GPE (16:0)	1.189	Lipid	Lysophospholipid
2-palmitoyl-GPE (16:0)*	1.373	Lipid	Lysophospholipid
1-margaroyl-GPE (17:0)*	1.003	Lipid	Lysophospholipid
1-stearoyl-GPE (18:0)	0.786	Lipid	Lysophospholipid
2-stearoyl-GPE (18:0)*	0.922	Lipid	Lysophospholipid
1-oleoyl-GPE (18:1)	1.154	Lipid	Lysophospholipid
2-oleoyl-GPE (18:1)*	1.093	Lipid	Lysophospholipid
1-linoleoyl-GPE (18:2)*	1.217	Lipid	Lysophospholipid
2-linoleoyl-GPE (18:2)*	0.979	Lipid	Lysophospholipid
1-linolenoyl-GPE (18:3)*	Not Scored	Lipid	Lysophospholipid
1-eicosenoyl-GPE (20:1)*	0.455	Lipid	Lysophospholipid
1-dihomo-linolenoyl-GPE (20:3n3 or 6)*	2.984	Lipid	Lysophospholipid
1-arachidonoyl-GPE (20:4n6)*	-0.216	Lipid	Lysophospholipid
2-arachidonoyl-GPE (20:4)*	-0.429	Lipid	Lysophospholipid
1-eicosapentaenoyl-GPE (20:5)*	2.934	Lipid	Lysophospholipid
1-adrenoyl-GPE (22:4)*	0.499	Lipid	Lysophospholipid
1-docosahexaenoyl-GPE (22:6)*	-0.725	Lipid	Lysophospholipid
1-oleoyl-GPG (18:1)*	Low filled	Lipid	Lysophospholipid
1-palmitoyl-GPI (16:0)	1.882	Lipid	Lysophospholipid
1-stearoyl-GPI (18:0)	1.075	Lipid	Lysophospholipid
2-stearoyl-GPI (18:0)*	0.921	Lipid	Lysophospholipid
1-oleoyl-GPI (18:1)	1.579	Lipid	Lysophospholipid
1-linoleoyl-GPI (18:2)*	0.433	Lipid	Lysophospholipid
1-arachidonoyl-GPI (20:4)*	0.014	Lipid	Lysophospholipid
1-palmityl-2-palmitoyl-GPC (O-16:0/16:0)*	-1.083	Lipid	Plasmalogen
1-palmityl-2-stearoyl-GPC (O-16:0/18:0)*	0.290	Lipid	Plasmalogen
1-palmityl-2-oleoyl-GPC (O-16:0/18:1)*	-0.049	Lipid	Plasmalogen
1-palmityl-2-linoleoyl-GPC (O-16:0/18:2)*	-2.003	Lipid	Plasmalogen
1-palmityl-2-arachidonoyl-GPC (O-16:0/20:4)*	-2.101	Lipid	Plasmalogen
1-stearyl-2-arachidonoyl-GPC (O-18:0/20:4)*	-2.679	Lipid	Plasmalogen
1-stearyl-2-docosapentaenoyl-GPC (O-18:0/22:5n3)*	0.046	Lipid	Plasmalogen
1-(1-enyl-oleoyl)-2-docosahexaenoyl-GPE (P-18:1/22:6)*	-1.701	Lipid	Plasmalogen
1-(1-enyl-palmitoyl)-2-docosahexaenoyl-GPC (P-16:0/22:6)*	-2.337	Lipid	Plasmalogen
1-(1-enyl-palmitoyl)-2-oleoyl-GPE (P-16:0/18:1)*	0.522	Lipid	Plasmalogen

1-(1-enyl-palmitoyl)-2-linoleoyl-GPE (P-16:0/18:2)*	-1.548	Lipid	Plasmalogen
1-(1-enyl-palmitoyl)-2-palmitoyl-GPC (P-16:0/16:0)*	-1.688	Lipid	Plasmalogen
1-(1-enyl-palmitoyl)-2-palmitoleoyl-GPC (P-16:0/16:1)*	-0.334	Lipid	Plasmalogen
1-(1-enyl-palmitoyl)-2-arachidonoyl-GPE (P-16:0/20:4)*	-3.432	Lipid	Plasmalogen
1-(1-enyl-palmitoyl)-2-docosahexaenoyl-GPE (P-16:0/22:6)*	-3.123	Lipid	Plasmalogen
1-(1-enyl-palmitoyl)-2-oleoyl-GPC (P-16:0/18:1)*	-0.997	Lipid	Plasmalogen
1-(1-enyl-stearoyl)-2-oleoyl-GPE (P-18:0/18:1)	0.446	Lipid	Plasmalogen
1-(1-enyl-stearoyl)-2-linoleoyl-GPE (P-18:0/18:2)*	-0.647	Lipid	Plasmalogen
1-(1-enyl-palmitoyl)-2-arachidonoyl-GPC (P-16:0/20:4)*	-3.157	Lipid	Plasmalogen
1-(1-enyl-palmitoyl)-2-linoleoyl-GPC (P-16:0/18:2)*	-3.485	Lipid	Plasmalogen
1-(1-enyl-stearoyl)-2-arachidonoyl-GPC (P-18:0/20:4)	-4.656	Lipid	Plasmalogen
1-(1-enyl-stearoyl)-2-arachidonoyl-GPE (P-18:0/20:4)*	-2.996	Lipid	Plasmalogen
1-(1-enyl-stearoyl)-2-docosahexaenoyl-GPC (P-18:0/22:6)*	-2.884	Lipid	Plasmalogen
1-(1-enyl-stearoyl)-2-docosahexaenoyl-GPE (P-18:0/22:6)*	-3.459	Lipid	Plasmalogen
1-(1-enyl-stearoyl)-2-docosapentaenoyl-GPE (P-18:0/22:5n3)*	-1.081	Lipid	Plasmalogen
1-(1-enyl-stearoyl)-2-dihomo-linolenoyl-GPE (P-18:0/20:3)*	0.333	Lipid	Plasmalogen
1-(1-enyl-stearoyl)-2-linoleoyl-GPC (P-18:0/18:2)*	-1.973	Lipid	Plasmalogen
1-(1-enyl-stearoyl)-2-oleoyl-GPC (P-18:0/18:1)	0.819	Lipid	Plasmalogen
phosphatidylcholine (O-18:1/20:4, O-16:0/22:5n3)*	-0.022	Lipid	Plasmalogen
1-palmityl-GPC (O-16:0)	0.070	Lipid	Lysoplasmalogen
1-stearyl-GPC (O-18:0)*	-0.263	Lipid	Lysoplasmalogen
1-(1-enyl-palmitoyl)-GPC (P-16:0)*	-0.385	Lipid	Lysoplasmalogen
1-(1-enyl-palmitoyl)-GPE (P-16:0)*	-1.049	Lipid	Lysoplasmalogen
1-(1-enyl-oleoyl)-GPC (P-18:1)*	0.158	Lipid	Lysoplasmalogen
1-(1-enyl-oleoyl)-GPE (P-18:1)*	-0.713	Lipid	Lysoplasmalogen
1-(1-enyl-stearoyl)-GPC (P-18:0)*	0.012	Lipid	Lysoplasmalogen
1-(1-enyl-stearoyl)-GPE (P-18:0)*	-1.136	Lipid	Lysoplasmalogen
glycerol	0.016	Lipid	Glycerolipid Metabolism
glycerol 3-phosphate	1.168	Lipid	Glycerolipid Metabolism
glycerophosphoglycerol	-0.141	Lipid	Glycerolipid Metabolism
1-palmitoylglycerol (16:0)	1.095	Lipid	Monoacylglycerol
1-palmitoleoylglycerol (16:1)*	Low filled	Lipid	Monoacylglycerol
1-oleoylglycerol (18:1)	0.587	Lipid	Monoacylglycerol
1-linoleoylglycerol (18:2)	-0.868	Lipid	Monoacylglycerol
1-linolenoylglycerol (18:3)	Low filled	Lipid	Monoacylglycerol
1-dihomo-linolenylglycerol (20:3)	Low filled	Lipid	Monoacylglycerol
1-arachidonylglycerol (20:4)	Low filled	Lipid	Monoacylglycerol
2-palmitoylglycerol (16:0)	0.394	Lipid	Monoacylglycerol
2-oleoylglycerol (18:1)	0.550	Lipid	Monoacylglycerol
diacylglycerol (16:1/18:2 [2], 16:0/18:3 [1])*	-1.391	Lipid	Diacylglycerol
palmitoyl-oleoyl-glycerol (16:0/18:1) [2]*	1.472	Lipid	Diacylglycerol
palmitoyl-linoleoyl-glycerol (16:0/18:2) [1]*	0.196	Lipid	Diacylglycerol

palmitoyl-linoleoyl-glycerol (16:0/18:2) [2]*	-0.301	Lipid	Diacylglycerol
palmitoyl-arachidonoyl-glycerol (16:0/20:4) [1]*	1.063	Lipid	Diacylglycerol
oleoyl-oleoyl-glycerol (18:1/18:1) [1]*	1.340	Lipid	Diacylglycerol
oleoyl-oleoyl-glycerol (18:1/18:1) [2]*	0.954	Lipid	Diacylglycerol
oleoyl-linoleoyl-glycerol (18:1/18:2) [1]	-0.275	Lipid	Diacylglycerol
oleoyl-linoleoyl-glycerol (18:1/18:2) [2]	-0.415	Lipid	Diacylglycerol
linoleoyl-linoleoyl-glycerol (18:2/18:2) [1]*	-3.329	Lipid	Diacylglycerol
linoleoyl-linolenoyl-glycerol (18:2/18:3) [2]*	0.617	Lipid	Diacylglycerol
oleoyl-arachidonoyl-glycerol (18:1/20:4) [1]*	0.590	Lipid	Diacylglycerol
oleoyl-arachidonoyl-glycerol (18:1/20:4) [2]*	0.308	Lipid	Diacylglycerol
linoleoyl-arachidonoyl-glycerol (18:2/20:4) [1]*	-0.853	Lipid	Diacylglycerol
linoleoyl-arachidonoyl-glycerol (18:2/20:4) [2]*	-1.325	Lipid	Diacylglycerol
sphinganine	-0.568	Lipid	Sphingolipid Synthesis
sphinganine-1-phosphate	0.184	Lipid	Sphingolipid Synthesis
N-palmitoyl-sphinganine (d18:0/16:0)	0.562	Lipid	Dihydroceramides
N-palmitoyl-sphingosine (d18:1/16:0)	0.505	Lipid	Ceramides
N-stearoyl-sphingosine (d18:1/18:0)*	2.150	Lipid	Ceramides
N-behenoyl-sphingadienine (d18:2/22:0)*	Low filled	Lipid	Ceramides
ceramide (d18:1/14:0, d16:1/16:0)*	0.234	Lipid	Ceramides
ceramide (d16:1/24:1, d18:1/22:1)*	Low filled	Lipid	Ceramides
ceramide (d18:2/24:1, d18:1/24:2)*	0.485	Lipid	Ceramides
glycosyl-N-palmitoyl-sphingosine (d18:1/16:0)	0.535	Lipid	Hexosylceramides (HCER)
glycosyl-N-stearoyl-sphingosine (d18:1/18:0)	1.168	Lipid	Hexosylceramides (HCER)
glycosyl-N-behenoyl-sphingadienine (d18:2/22:0)*	-0.997	Lipid	Hexosylceramides (HCER)
glycosyl ceramide (d18:1/20:0, d16:1/22:0)*	0.343	Lipid	Hexosylceramides (HCER)
glycosyl ceramide (d18:2/24:1, d18:1/24:2)*	0.379	Lipid	Hexosylceramides (HCER)
lactosyl-N-palmitoyl-sphingosine (d18:1/16:0)	0.053	Lipid	Lactosylceramides (LCER)
lactosyl-N-behenoyl-sphingosine (d18:1/22:0)*	-1.110	Lipid	Lactosylceramides (LCER)
lactosyl-N-nervonoyl-sphingosine (d18:1/24:1)*	1.277	Lipid	Lactosylceramides (LCER)
myristoyl dihydrosphingomyelin (d18:0/14:0)*	1.022	Lipid	Sphingomyelins
palmitoyl dihydrosphingomyelin (d18:0/16:0)*	0.087	Lipid	Sphingomyelins
behenoyl dihydrosphingomyelin (d18:0/22:0)*	-1.242	Lipid	Sphingomyelins
sphingomyelin (d18:0/18:0, d19:0/17:0)*	0.003	Lipid	Sphingomyelins
sphingomyelin (d18:0/20:0, d16:0/22:0)*	-0.869	Lipid	Sphingomyelins
palmitoyl sphingomyelin (d18:1/16:0)	0.490	Lipid	Sphingomyelins
stearoyl sphingomyelin (d18:1/18:0)	-0.306	Lipid	Sphingomyelins
behenoyl sphingomyelin (d18:1/22:0)*	0.882	Lipid	Sphingomyelins
tricosanoyl sphingomyelin (d18:1/23:0)*	-0.894	Lipid	Sphingomyelins

lignoceroyl sphingomyelin (d18:1/24:0)	-0.159	Lipid	Sphingomyelins
sphingomyelin (d18:2/18:1)*	-2.585	Lipid	Sphingomyelins
sphingomyelin (d18:2/23:1)*	-1.052	Lipid	Sphingomyelins
sphingomyelin (d18:2/24:2)*	-1.774	Lipid	Sphingomyelins
sphingomyelin (d17:1/14:0, d16:1/15:0)*	-0.074	Lipid	Sphingomyelins
sphingomyelin (d18:1/14:0, d16:1/16:0)*	0.405	Lipid	Sphingomyelins
sphingomyelin (d18:2/14:0, d18:1/14:1)*	0.162	Lipid	Sphingomyelins
sphingomyelin (d17:1/16:0, d18:1/15:0, d16:1/17:0)*	-0.916	Lipid	Sphingomyelins
sphingomyelin (d17:2/16:0, d18:2/15:0)*	-0.394	Lipid	Sphingomyelins
sphingomyelin (d18:2/16:0, d18:1/16:1)*	-0.571	Lipid	Sphingomyelins
sphingomyelin (d18:1/17:0, d17:1/18:0, d19:1/16:0)	-1.109	Lipid	Sphingomyelins
sphingomyelin (d18:1/18:1, d18:2/18:0)	-0.332	Lipid	Sphingomyelins
sphingomyelin (d18:1/19:0, d19:1/18:0)*	-0.739	Lipid	Sphingomyelins
sphingomyelin (d18:1/20:0, d16:1/22:0)*	-1.360	Lipid	Sphingomyelins
sphingomyelin (d18:1/20:1, d18:2/20:0)*	-1.531	Lipid	Sphingomyelins
sphingomyelin (d18:1/20:2, d18:2/20:1, d16:1/22:2)*	-2.778	Lipid	Sphingomyelins
sphingomyelin (d18:1/21:0, d17:1/22:0, d16:1/23:0)*	-0.929	Lipid	Sphingomyelins
sphingomyelin (d18:2/21:0, d16:2/23:0)*	-1.050	Lipid	Sphingomyelins
sphingomyelin (d18:1/22:1, d18:2/22:0, d16:1/24:1)*	-1.582	Lipid	Sphingomyelins
sphingomyelin (d18:1/22:2, d18:2/22:1, d16:1/24:2)*	-1.929	Lipid	Sphingomyelins
sphingomyelin (d18:2/23:0, d18:1/23:1, d17:1/24:1)*	-0.653	Lipid	Sphingomyelins
sphingomyelin (d18:1/24:1, d18:2/24:0)*	3.243	Lipid	Sphingomyelins
sphingomyelin (d18:2/24:1, d18:1/24:2)*	-0.162	Lipid	Sphingomyelins
sphingomyelin (d18:1/25:0, d19:0/24:1, d20:1/23:0, d19:1/24:0)*	-3.043	Lipid	Sphingomyelins
sphingosine	-1.133	Lipid	Sphingosines
sphingosine 1-phosphate	0.151	Lipid	Sphingosines
3-hydroxy-3-methylglutarate	0.231	Lipid	Mevalonate Metabolism
cholesterol	0.371	Lipid	Sterol
7alpha-hydroxy-3-oxo-4-cholestenoate (7-Hoca)	-0.546	Lipid	Sterol
3beta-hydroxy-5-cholestenoate	-1.977	Lipid	Sterol
4-cholesten-3-one	0.809	Lipid	Sterol
campesterol	Low filled	Lipid	Sterol
pregnenolone sulfate	-1.006	Lipid	Pregnenolone Steroids
21-hydroxypregnenolone disulfate	0.138	Lipid	Pregnenolone Steroids
pregnenediol sulfate (C21H34O5S)*	-1.434	Lipid	Pregnenolone Steroids
pregnenediol disulfate (C21H34O8S2)*	-0.010	Lipid	Pregnenolone Steroids
5alpha-pregnan-3beta,20alpha-diol disulfate	-2.704	Lipid	Progestin Steroids
cortisol	-3.201	Lipid	Corticosteroids
dehydroepiandrosterone sulfate (DHEA-S)	-1.462	Lipid	Androgenic Steroids
16alpha-hydroxy DHEA 3-sulfate	0.338	Lipid	Androgenic Steroids

androstenediol (3beta,17beta) disulfate (1)	-0.622	Lipid	Androgenic Steroids
androstenediol (3beta,17beta) disulfate (2)	-0.716	Lipid	Androgenic Steroids
andro steroid monosulfate C19H28O6S (1)*	0.624	Lipid	Androgenic Steroids
cholate	-2.738	Lipid	Primary Bile Acid Metabolism
glycocholate	-1.266	Lipid	Primary Bile Acid Metabolism
taurocholate	-1.155	Lipid	Primary Bile Acid Metabolism
chenodeoxycholate	-2.233	Lipid	Primary Bile Acid Metabolism
glycochenodeoxycholate	-1.432	Lipid	Primary Bile Acid Metabolism
taurochenodeoxycholate	-1.360	Lipid	Primary Bile Acid Metabolism
tauro-beta-muricholate	1.194	Lipid	Primary Bile Acid Metabolism
glyco-beta-muricholate**	1.539	Lipid	Primary Bile Acid Metabolism
glycochenodeoxycholate glucuronide (1)	-0.562	Lipid	Primary Bile Acid Metabolism
glycochenodeoxycholate 3-sulfate	-1.162	Lipid	Primary Bile Acid Metabolism
deoxycholate	0.543	Lipid	Secondary Bile Acid Metabolism
glycodeoxycholate	0.780	Lipid	Secondary Bile Acid Metabolism
taurodeoxycholate	0.259	Lipid	Secondary Bile Acid Metabolism
glycolithocholate	1.090	Lipid	Secondary Bile Acid Metabolism
glycolithocholate sulfate*	1.141	Lipid	Secondary Bile Acid Metabolism
taurolithocholate 3-sulfate	0.787	Lipid	Secondary Bile Acid Metabolism
isoursodeoxycholate	-0.621	Lipid	Secondary Bile Acid Metabolism
glycoursodeoxycholate	-0.801	Lipid	Secondary Bile Acid Metabolism
hyocholate	0.198	Lipid	Secondary Bile Acid Metabolism
glycohyocholate	-0.211	Lipid	Secondary Bile Acid Metabolism
glycocholenate sulfate*	-1.824	Lipid	Secondary Bile Acid Metabolism
taurocholenate sulfate*	-1.470	Lipid	Secondary Bile Acid Metabolism
glycodeoxycholate 3-sulfate	0.670	Lipid	Secondary Bile Acid Metabolism
inosine	-0.786	Nucleotide	Purine Metabolism, (Hypo)Xanthine/Inosine containing

hypoxanthine	0.506	Nucleotide	Purine Metabolism, (Hypo)Xanthine/Inosine containing
xanthine	-0.802	Nucleotide	Purine Metabolism, (Hypo)Xanthine/Inosine containing
N1-methylinosine	0.709	Nucleotide	Purine Metabolism, (Hypo)Xanthine/Inosine containing
urate	-1.835	Nucleotide	Purine Metabolism, (Hypo)Xanthine/Inosine containing
allantoin	-1.190	Nucleotide	Purine Metabolism, (Hypo)Xanthine/Inosine containing
adenosine 5'-monophosphate (AMP)	-1.869	Nucleotide	Purine Metabolism, Adenine containing
adenosine	-0.270	Nucleotide	Purine Metabolism, Adenine containing
adenine	-0.415	Nucleotide	Purine Metabolism, Adenine containing
N1-methyladenosine	1.713	Nucleotide	Purine Metabolism, Adenine containing
N6-carbamoylthreonyladenosine	0.626	Nucleotide	Purine Metabolism, Adenine containing
N6-succinyladenosine	0.135	Nucleotide	Purine Metabolism, Adenine containing
7-methylguanine	-0.052	Nucleotide	Purine Metabolism, Guanine containing
N2,N2-dimethylguanosine	1.580	Nucleotide	Purine Metabolism, Guanine containing
N-carbamoylaspartate	-1.197	Nucleotide	Pyrimidine Metabolism, Orotate containing

orotate	0.041	Nucleotide	Pyrimidine Metabolism, Orotate containing
orotidine	0.504	Nucleotide	Pyrimidine Metabolism, Orotate containing
uridine	-1.309	Nucleotide	Pyrimidine Metabolism, Uracil containing
pseudouridine	0.514	Nucleotide	Pyrimidine Metabolism, Uracil containing
5,6-dihydrouridine	-0.259	Nucleotide	Pyrimidine Metabolism, Uracil containing
2'-O-methyluridine	0.105	Nucleotide	Pyrimidine Metabolism, Uracil containing
5-methyluridine (ribothymidine)	1.431	Nucleotide	Pyrimidine Metabolism, Uracil containing
2'-deoxyuridine	Not Scored	Nucleotide	Pyrimidine Metabolism, Uracil containing
3-ureidopropionate	-0.445	Nucleotide	Pyrimidine Metabolism, Uracil containing
beta-alanine	-2.234	Nucleotide	Pyrimidine Metabolism, Uracil containing
N-acetyl-beta-alanine	1.060	Nucleotide	Pyrimidine Metabolism, Uracil containing
3-methylcytidine	Not Scored	Nucleotide	Pyrimidine Metabolism, Cytidine containing
N4-acetylcytidine	0.170	Nucleotide	Pyrimidine Metabolism, Cytidine containing
2'-O-methylcytidine	Not Scored	Nucleotide	Pyrimidine Metabolism, Cytidine containing
5,6-dihydrothymine	1.618	Nucleotide	Pyrimidine Metabolism, Thymine containing
3-aminoisobutyrate	-0.018	Nucleotide	Pyrimidine Metabolism, Thymine containing
quinolinate	0.867	Cofactors and Vitamins	Nicotinate and Nicotinamide Metabolism
nicotinamide	-0.402	Cofactors and Vitamins	Nicotinate and Nicotinamide Metabolism

1-methylnicotinamide	-1.136	Cofactors and Vitamins	Nicotinate and Nicotinamide Metabolism
trigonelline (N'-methylnicotinate)	-3.444	Cofactors and Vitamins	Nicotinate and Nicotinamide Metabolism
N1-methyl-2-pyridone-5-carboxamide	1.011	Cofactors and Vitamins	Nicotinate and Nicotinamide Metabolism
pantothenate	-0.142	Cofactors and Vitamins	Pantothenate and CoA Metabolism
threonate	0.685	Cofactors and Vitamins	Ascorbate and Aldarate Metabolism
oxalate (ethanedioate)	1.287	Cofactors and Vitamins	Ascorbate and Aldarate Metabolism
gulonate*	Not Scored	Cofactors and Vitamins	Ascorbate and Aldarate Metabolism
alpha-tocopherol	1.362	Cofactors and Vitamins	Tocopherol Metabolism
gamma-CEHC	0.305	Cofactors and Vitamins	Tocopherol Metabolism
gamma-CEHC glucuronide*	Low filled	Cofactors and Vitamins	Tocopherol Metabolism
delta-CEHC	-0.159	Cofactors and Vitamins	Tocopherol Metabolism
gamma-tocopherol/beta-tocopherol	0.094	Cofactors and Vitamins	Tocopherol Metabolism
heme	0.822	Cofactors and Vitamins	Hemoglobin and Porphyrin Metabolism
bilirubin (Z,Z)	-1.744	Cofactors and Vitamins	Hemoglobin and Porphyrin Metabolism
bilirubin (E,E)*	-0.735	Cofactors and Vitamins	Hemoglobin and Porphyrin Metabolism
bilirubin (E,Z or Z,E)*	-1.960	Cofactors and Vitamins	Hemoglobin and Porphyrin Metabolism
biliverdin	-2.139	Cofactors and Vitamins	Hemoglobin and Porphyrin Metabolism
retinol (vitamin A)	1.424	Cofactors and Vitamins	Vitamin A Metabolism
carotene diol (1)	-2.749	Cofactors and Vitamins	Vitamin A Metabolism
carotene diol (2)	-3.265	Cofactors and Vitamins	Vitamin A Metabolism
pyridoxal	-0.659	Cofactors and Vitamins	Vitamin B6 Metabolism
pyridoxate	1.225	Cofactors and Vitamins	Vitamin B6 Metabolism
hippurate	-0.573	Xenobiotics	Benzoate Metabolism

2-hydroxyhippurate (salicylurate)	-0.782	Xenobiotics	Benzoate Metabolism
4-hydroxyhippurate	0.872	Xenobiotics	Benzoate Metabolism
benzoate	2.741	Xenobiotics	Benzoate Metabolism
catechol sulfate	2.405	Xenobiotics	Benzoate Metabolism
4-methylguaiacol sulfate	1.774	Xenobiotics	Benzoate Metabolism
guaiacol sulfate	2.641	Xenobiotics	Benzoate Metabolism
3-methyl catechol sulfate (1)	-0.540	Xenobiotics	Benzoate Metabolism
4-methylcatechol sulfate	1.646	Xenobiotics	Benzoate Metabolism
4-ethylphenylsulfate	-1.677	Xenobiotics	Benzoate Metabolism
4-vinylphenol sulfate	-2.693	Xenobiotics	Benzoate Metabolism
3-methoxycatechol sulfate (1)	-1.355	Xenobiotics	Benzoate Metabolism
methyl-4-hydroxybenzoate sulfate	2.068	Xenobiotics	Benzoate Metabolism
propyl 4-hydroxybenzoate sulfate	1.432	Xenobiotics	Benzoate Metabolism
p-cresol sulfate	0.636	Xenobiotics	Benzoate Metabolism
theobromine	-1.522	Xenobiotics	Xanthine Metabolism
2-piperidinone	-1.275	Xenobiotics	Food Component/Plant
sucralose	Not Scored	Xenobiotics	Food Component/Plant
2,3-dihydroxyisovalerate	-0.647	Xenobiotics	Food Component/Plant
2-isopropylmalate	-0.576	Xenobiotics	Food Component/Plant
gluconate	-1.576	Xenobiotics	Food Component/Plant
erythritol	1.169	Xenobiotics	Food Component/Plant
homostachydrine*	-4.105	Xenobiotics	Food Component/Plant
N-(2-furoyl)glycine	0.733	Xenobiotics	Food Component/Plant
phytanate	-1.540	Xenobiotics	Food Component/Plant
saccharin	1.229	Xenobiotics	Food Component/Plant
acesulfame	1.379	Xenobiotics	Food Component/Plant
stachydrine	-3.090	Xenobiotics	Food Component/Plant
tartarate	Not Scored	Xenobiotics	Component/Plant

pyrraline	1.079	Xenobiotics	Food Component/Plant
furaneol sulfate	Not Scored	Xenobiotics	Food Component/Plant
tartronate (hydroxymalonate)	-0.697	Xenobiotics	Food Component/Plant
2-aminophenol sulfate	-0.692	Xenobiotics	Food Component/Plant
salicylate	-1.869	Xenobiotics	Drug - Topical Agents
sulfate*	0.019	Xenobiotics	Chemical
O-sulfo-tyrosine	0.300	Xenobiotics	Chemical
iminodiacetate (IDA)	-1.916	Xenobiotics	Chemical
perfluorooctanesulfonate (PFOS)	-2.418	Xenobiotics	Chemical
4-hydroxychlorothalonil	-2.942	Xenobiotics	Chemical
1,2,3-benzenetriol sulfate (2)	-0.330	Xenobiotics	Chemical
3-hydroxypyridine sulfate	-0.555	Xenobiotics	Chemical
6-hydroxyindole sulfate	0.544	Xenobiotics	Chemical
perfluorooctanoate (PFOA)	-2.413	Xenobiotics	Chemical
glycine conjugate of C10H14O2 (1)*	-0.770	Partially Characterized Molecules	Partially Characterized Molecules
glutamine_degradant*	0.545	Partially Characterized Molecules	Partially Characterized Molecules
thiamin (Vitamin B1)	rare	Cofactors and Vitamins	Thiamine Metabolism
5,6-dihydrouracil	rare	Nucleotide	Pyrimidine Metabolism, Uracil containing
formiminoglutamate	rare	Amino Acid	Histidine Metabolism
cis-urocanate	rare	Amino Acid	Histidine Metabolism
tigloylglycine	rare	Amino Acid	Leucine, Isoleucine and Valine Metabolism
2-methylbutyrylglycine	rare	Amino Acid	Leucine, Isoleucine and Valine Metabolism
beta-hydroxyisovaleroylcarnitine	rare	Amino Acid	Leucine, Isoleucine and Valine Metabolism
ribose	rare	Carbohydrate	Pentose Metabolism
palmitoleoyl-linoleoyl-glycerol (16:1/18:2) [1]*	rare	Lipid	Diacylglycerol
butyrylglycine	rare	Lipid	Fatty Acid Metabolism (also BCAA Metabolism)
dihydroorotate	rare	Nucleotide	Pyrimidine Metabolism,Orotate containing
gamma-glutamylalanine	rare	Peptide	Gamma-glutamyl Amino Acid

thioproline	rare	Xenobiotics	Chemical
